# Laser Acupuncture: Two Acupoints (Baihui, Neiguan) and Two Modalities of Laser (658 nm, 405 nm) Induce Different Effects in Neurovegetative Parameters

**DOI:** 10.1155/2013/432764

**Published:** 2013-06-03

**Authors:** Gerhard Litscher, Lu Wang, Xiaoyu Wang, Ingrid Gaischek

**Affiliations:** ^1^Stronach Research Unit for Complementary and Integrative Laser Medicine, Research Unit of Biomedical Engineering in Anesthesia and Intensive Care Medicine, TCM Research Center Graz, Medical University of Graz, Auenbruggerplatz 29, 8036 Graz, Austria; ^2^Institute of Acupuncture and Moxibustion, China Academy of Chinese Medical Sciences, Beijing 100700, China

## Abstract

There are only few scientific publications dealing with the basic investigation of the effects of only one or two acupoints or comparing one single point with another single point, using different stimulation methods in the same persons. The aim of this needle-controlled, randomized crossover study was to investigate the neurovegetative parameters heart rate (HR) and heart rate variability (HRV) using two different acupoints, Baihui (GV20) and Neiguan (PC6), in separate sessions. We investigated 11 healthy volunteers (3 m, 8 f) with a mean age ± SD of 22.9 ± 2.8 years. The two acupoints were stimulated for 10 minutes each with manual needle acupuncture, red laser acupuncture (658 nm), and violet laser acupuncture (405 nm), in randomized order. Needle and red laser stimulation of the Baihui acupoint decreased HR significantly. Only violet laser stimulation at the Neiguan acupoint induced a significant increase of total HRV. Further studies using other neurovegetative parameters and more volunteers are necessary to confirm the preliminary results.

## 1. Introduction

The term “laser” is often connected with precision, future, and innovation. Only very few other areas have shown such a rapid and enormous development over the last years as medical laser applications. High-tech laser acupuncture [1], intravenous laser blood irradiation [2], and noninvasive and invasive laser needle stimulation [1–3] are only some of the future-oriented laser therapy options in medicine.

Our research group was the first to investigate violet laser acupuncture (405 nm). Important contributions on this topic were published between 2010 and 2012 [4–8]. The most important difference between violet and, for example, red laser (658 nm) is the penetration depth (405 nm: 1-2 mm; 658 nm: 3-4 cm) on the one hand and the fact that the entire energy of the violet laser is absorbed already at the skin surface on the other hand. In practical terms, this means that the red laser cannot be felt, whereas the violet laser can, thus inducing a stronger deqi sensation [9].

The aim of this needle-controlled, randomized crossover study was to investigate neurovegetative parameters like heart rate (HR) and heart rate variability (HRV) in two different sessions using two different acupoints in healthy volunteers. 

## 2. Materials and Methods

### 2.1. Stimulation Methods

#### 2.1.1. Manual Needle Acupuncture

Needle acupuncture was performed with single-use sterile needles (0.30 × 30 mm, Huan Qiu, Suzhou, China). After disinfecting the skin at the chosen acupoint(s) (see below), the needle was inserted and stimulated clockwise and counterclockwise for 15 seconds each, with two rotations per second, resulting in 30 rotations [10]. After ten minutes, the needle was removed.

#### 2.1.2. Red Laser

**Figure 1:**
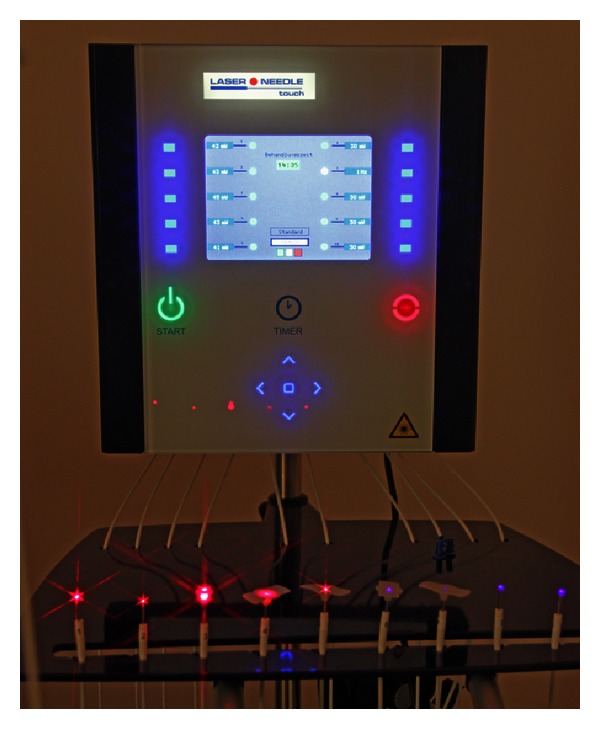
Laser needle system at the Medical University of Graz.

The red laser needle radiation (658 nm) was coupled into optical fibers, and the laser needles were arranged at distal ends of the optical fibers of a Laserneedle-touch system (Laserneedle GmbH, Berlin, Germany; see Figure 1). Output power of each laser needle was 40 mW. The fiber core diameter was about 500 *μ*m. The time of irradiation was 10 minutes, resulting in an energy density of about 20 J/cm² per acupoint. A continuous wave (cw) mode was applied. The needles were placed vertically on the skin using special applicators, triggering painless and nonperceptible stimulation at the acupoint. The method is described in detail in previous publications [11].

#### 2.1.3. Violet Laser

Noninvasive violet optical laser needles (wavelength: 405 nm, output power 110 mW, laser needle spot diameter 500 *μ*m, duration: 10 min, cw-mode) were also fixed onto the skin, but not inserted. The same laser needle system described above was used. Optical energy density was very high (range: kJ/cm²). More details concerning the technical parameters of laser needle acupuncture can also be found in recent publications [12].

### 2.2. Neurovegetative Monitoring

The two neurovegetative parameters were recorded using an HRV Medilog AR12 (Huntleigh Healthcare, Cardiff, UK; and Leupamed GmbH, Graz, Austria) system. This system is designed for a monitoring period up to 24 hours. The sampling rate of the recorder is 4096 Hz, so that R waves can be detected extremely accurately. Three “Skintact Premier F-55” ECG electrodes (Leonhard Lang GmbH, Innsbruck, Austria) were fixed on the chest. All raw data are stored digitally on special memory cards. 

HRV is measured as the percentage change in sequential chamber complexes (RR intervals) in the ECG. HRV can be quantified over time using registration of percentage changes in RR intervals in the time domain as well as the changes in the frequency range by analysis of electrocardiographic power spectra. Parameters are recommended by the task force of the European Society of Cardiology and the North American Society of Pacing and Electrophysiology [13]. Calculation of ECG power spectra is thought to provide an understanding of the effects of sympathetic and parasympathetic systems on HRV and is used also in acupuncture research [13–23]. Early work pointed out a few bands in the spectrum of HRV that could be interpreted as markers of physiological relevance. Associated mechanisms are thermoregulation which can be found in the very low frequency band, blood pressure and respiratory effects [13]. To the best of our knowledge, there are no scientific results concerning laser acupuncture and long-lasting thermoregulatory effects which might be reflected in HRV.

### 2.3. Volunteers

The investigations were performed in eleven healthy volunteers (M/F, 3/8) with a mean age ± SD of 22.9 ± 2.8 years. Body height was 172.9 ± 7.4 cm and body weight 66.0 ± 10.4 kg. None of the subjects was under the influence of any medication. The registration of the non-invasive parameters was approved by the local ethics committee and in accordance with the Declaration of Helsinki of the World Medical Association. All persons provided written informed consent.

### 2.4. Acupuncture Points

**Figure 2:**
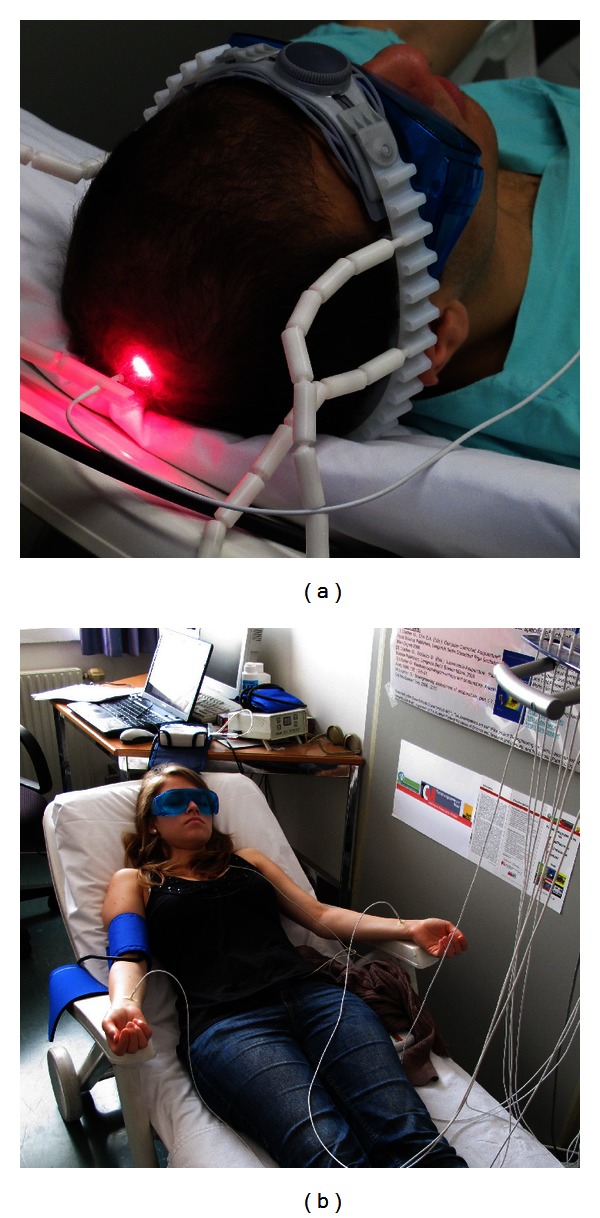
Acupuncture points Baihui (GV20; (a)) and Neiguan (PC6; (b)).

The following acupuncture points were used in our study: Baihui (GV20) and Neiguan (PC6) (see Figures 2(a) and 2(b)). Baihui is one of the most important acupoints of the Du meridian (governing vessel) and commonly used in neurology and psychiatry [24]. It is located on the continuation of the line connecting the lowest and highest points of the ear, on the median line of the head, 7 cun above the posterior hairline, and 5 cun behind the anterior hairline [25]. Neiguan is located on the palmar side of the forearm, on the line connecting Quze (PC3) and Daling (PC7), 2 cun proximal to the transverse crease of the wrist, and between the tendons of m. palmaris longus and m. flexor carpi radialis [26]. This point was stimulated bilaterally (see Figure 2(b)). Its main indications are cardiac pain, palpitation, oppressed feeling on the chest, vomiting, epilepsy, mania, and febrile diseases.

### 2.5. Measurement Procedure

The procedure was divided into three parts: violet laser, red laser, or needle acupuncture, which were performed in the same subjects in a randomized order. Between the different stimulation modalities, there was a break of at least 10 min. Each of the acupoints described above was tested in all subjects in separate sessions on two different days (cross-over), also in randomized order. The participants were lying comfortably on a bed during the entire investigation.

**Figure 3:**
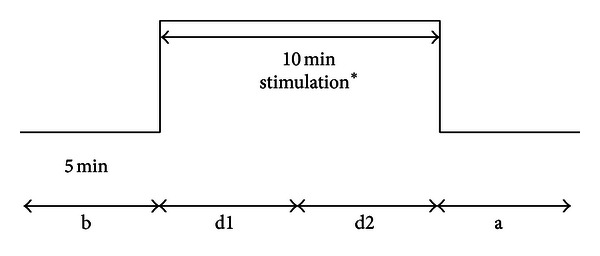
Recording profile. *Manual needle stimulation, red laser (658 nm), and violet laser (405 nm), in randomized order. (b) before stimulation; (d1, d2), two phases during stimulation; (a) after stimulation.

The measurement procedure, and the 5-minute segments (altogether 20 min) are shown in Figure 3.

### 2.6. Statistical Analysis

Data were analyzed with one-way repeated measures analysis of variance (ANOVA; SigmaPlot 12.0, Systat Software, Chicago, IL, USA), and the Tukey test was used for *post hoc* analysis. The level of significance was defined as *P* < 0.05.

## 3. Results

**Figure 4:**
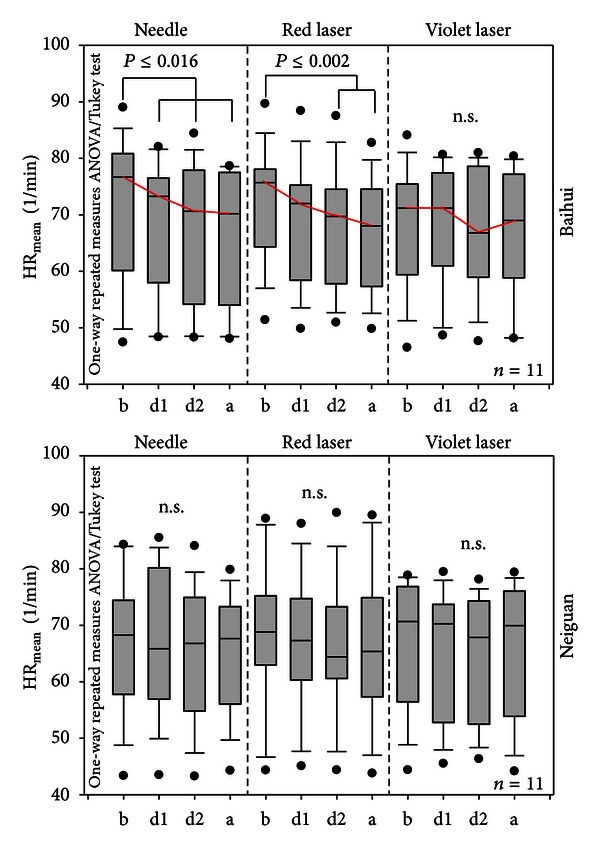
Box plot presentation of HR results. Note the significant decrease of HR (needle and red laser acupuncture). The lines in the boxes represent the median; the ends of the boxes, the 25th and 75th percentile; the error bars, the 10th and 90th percentile; and the dots, the outliers. (b) Before stimulation; (d1, d2), two phases during stimulation; (a) after stimulation.

Figure 4 shows HR values from the measurements. During and after stimulation of the Baihui acupoint, HR decreased; however, the effect was significant only during and after needle and red laser stimulation. Stimulation of Neiguan did not induce any significant changes in HR.

**Figure 5:**
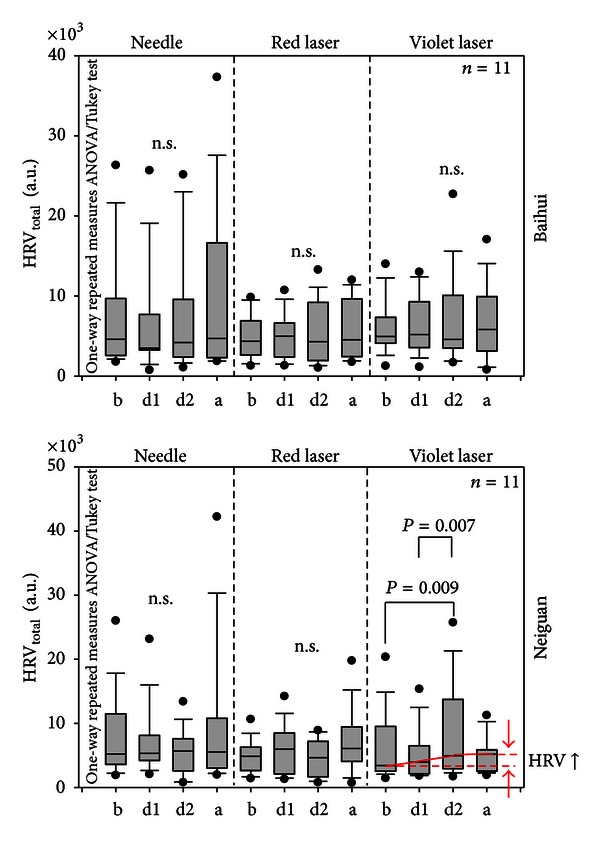
Changes in total HRV. A significant increase occurred only during violet laser stimulation of the Neiguan acupoint. For further explanations, see Figure 4.

The values of total HRV of all 11 healthy volunteers are summarized in Figure 5. Neither needle nor red laser nor violet laser stimulation at the Baihui acupoint induced significant changes in HRV, although HR decreased during and after all three stimulation modalities (cf. Figure 4). However, violet laser stimulation of the Neiguan acupoints showed a significant increase during the second half of the stimulation phase (d2) in comparison to the baseline values (b) and also to the first half of the stimulation phase (d1). It is interesting that needle and red laser stimulation did not induce similar effects.

## 4. Discussion

Acupuncture, an orientalmedicine technique that can be traced back at least 2,500 years, is gaining popularity as an alternative and complementary intervention in the Western world. According to traditional Chinese medicine (TCM), acupoints are distributed along meridians beneath the body's surface [27]. In TCM, the single acupuncture points will often have different effects, although they are used in the treatment of the same disease. When investigating the effectiveness of acupuncture on certain diseases, acupoint combinations or schemes are usually used. There are only few scientific publications dealing with the basic investigation of the effects of only one or two acupoints. There are even fewer studies comparing one single point with another single point, using different stimulation methods in the same persons, as performed in this study.

Baihui (GV20) is one of the most important acupoints of the entire meridian acupoint system. It was reported that electroacupuncture stimulation at GV20 increased the cerebral perfusion in the cerebral cortex which was suppressed in endothelial nitric oxide synthase knockout mice [28] and also increased cerebral blood flow in a model of ischemic brain injury in rats [29]. There were also reports of acupuncture stimulation of GV20 working well in lowering blood pressure not only in hypertension patients [30] and healthy subjects [31] but also in spontaneously hypertensive rats [32]. Similar effects may be relevant for decreasing abnormally elevated glutamate and acetylcholine levels in the lesioned side of the striatum [33]. Another aspect of this study on the effect of GV20 was that the point was ascribed the ability to calm and stabilize emotions [33]. In the clinical procedure for patients with sleep disturbances, Baihui is the most commonly used acupuncture point [34, 35]. Acupuncture on GV20 mitigated the anxiety symptoms of women undergoing in vitro fertilization [36] and patients during dental treatment [37]. GV20 was found to have the ability of vasodilatation and reduction of sympathetic activity in the stress response [28–32]. In the present study, we found GV20 to have the effect of decreasing HR, which is in accordance with the previously mentioned effects.

Neiguan (PC6) is also a classic acupuncture point in TCM. It is considered to be effective when treating cardiovascular disorders. Accumulating scientific evidence has recently shown that PC6 could modulate cardiovascular functions, possibly through activation of the rostral ventrolateral medullar (RVLM) area [38, 39]. It was also reported that stimulation of PC6 decreased the extent of myocardial ischemia by means of reducing the myocardial oxygen demand in animals, and it reduced sympathoexcitatory cardiovascular reflex responses, partly through an effect on the RVLM [40, 41]. Another study suggested that PC6 and ST36 both affected cardiac activities in healthy volunteers [42]. In reports from patients suffering from circadian rhythm disorders, laser acupuncture stimulation applied on PC6 increased vagal activity and suppression of cardiac sympathetic nerves [43]. However, another study suggested that HRV was not influenced by laser needle acupuncture at the Neiguan point (PC6) [44]. This is similar to the observations in our experiment. Further studies concerning possible long-term effects of different kinds of lasers on neurovegetative parameters are desirable.

## 5. Conclusions

The following conclusions can be drawn from the results of this study.

- Needle and red laser stimulation of the Baihui acupoint decrease heart rate in human subjects significantly.
- Only violet laser stimulation at the Neiguan acupoint induces a significant increase of total heart rate variability. This is even more interesting because of the fact that at the same time HR did not change significantly.
